# Effects of a home visiting nurse intervention versus care as usual on individual activities of daily living: a secondary analysis of a randomized controlled trial

**DOI:** 10.1186/1471-2318-14-24

**Published:** 2014-02-20

**Authors:** Bruce Friedman, Yanen Li, Dianne V Liebel, Bethel A Powers

**Affiliations:** 1 Department of Public Health Sciences, University of Rochester, 265 Crittenden Blvd, CU 420644, Rochester, NY 14642, USA; 2 School of Nursing, University of Rochester, 601 Elmwood Avenue, Box SON Rochester, NY 14642, USA

**Keywords:** Activities of daily living, Disability, Home care, Visiting nurses, Medicare

## Abstract

**Background:**

Home visiting nurses (HVNs) have long been part of home and community-based care interventions designed to meet the needs of functionally declining older adults. However, only one of the studies including HVNs that have demonstrated successful impacts on Activities of Daily Living (ADL) has reported how those interventions affected individual ADLs such as bathing, instead reporting the effect on means of various ADL indices and scales. Reporting impacts on means is insufficient since the same mean can consist of many different combinations of individual ADL impairments. The purpose of our study was to identify which individual ADLs were affected by a specific HVN intervention.

**Methods:**

This is a secondary analysis comparing two arms of a randomized controlled study that enrolled Medicare patients (mean age = 76.8 years; 70% female) with considerable ADL impairment. At baseline difficulty with individual ADLs ranged from a low of 16.0% with eating to a high of 78.0% with walking. Through monthly home visits, the HVN focused on empowering patients and using behavior change approaches to facilitate chronic disease self-management. Three categories of analyses were used to compare difficulty with and dependence in 6 individual ADLs between the HVN (n = 237) and care as usual (n = 262) groups (total N = 499) at 22 months after study entry: (1) unadjusted analyses that strictly depend on random assignment, (2) multinomial logistic regression analyses adjusting for baseline risk factors, and (3) multinomial regression analyses that include variables reporting post-randomization healthcare use as well as the baseline risk factors.

**Results:**

Compared to care as usual, patients receiving the HVN intervention had less difficulty performing bathing at 22 months. However, there were no effects for difficulty performing the other 5 ADLs. While no effects were found for lower levels of dependence for any ADLs, impacts were detected for the most dependent levels of 4 ADLs: patients experienced less dependence in walking and transferring, a substitution effect for toileting, and more dependence in eating.

**Conclusions:**

Future research is needed to confirm these findings and determine how HVN interventions affect individual ADLs of older adults with multiple ADLs.

## Background

Beginning in the 1960s, a variety of interventions have been developed that aim to better meet the needs of older persons whose physical or mental functioning has declined yet their desire is to continue living in the community rather than relocate to a nursing home or other institution [1-6]. Many of these interventions had as a goal the improvement or prevention of further declines in activities of daily living (ADL), which are basic tasks essential to everyday life, for example, bathing and dressing [7]. While a large number of studies have examined the effect of interventions on ADL among older adults, a considerably smaller number of these studies have been found to be effective, that is, have improved ADL, prevented further ADL decline, or slowed the rate of decline. Home visiting nurses (HVNs) have participated in 13 interventions with successful impacts on ADL [8-20]. These 13 interventions are extremely heterogeneous. Only two consisted exclusively of home visiting nursing [8,9], and one of these almost completely focused on informal caregivers rather than directly on patients [9]. The other 11 interventions included components in addition to home visiting nursing, namely, formal multidisciplinary conferences [10-14], social worker case management [12,15], collaboration with geriatricians [11,14,16], a community geriatric evaluation unit [11], community-based long term care [17], a health center provider team [18], reablement home care staff, home care managers, occupational therapists, and other categories of healthcare professionals [19], and a multi-component restorative program that is also separately delivered by a physiotherapist or an occupational therapist [20].

All but one of the HVN studies that report effective ADL findings *do not report* how the intervention affects *individual* ADLs. Rather, these studies present information on the Katz ADL Index [8,10,15], Barthel-formatted ADL items comprising scales with ranges of 0–20 [14] and 0–100 [9,18], an ADL scale ranging from 9 to 29 based on modified Barthel items [20], ADL scales with ranges of 0–100 [16], 0–6 [11], 0–20 [17], 5–15 [12], and 0–12 (OASIS-B items) [13], and the proportion of subjects dependent in at least one ADL [16]. The one exception [19] included 11 ADLs for which both a total mean and individual item data are presented.

It is critical to know which individual ADLs are successfully affected by HVN interventions and which are not because large numbers of older persons with ADL difficulty or dependence receive home healthcare, for example, each day about 850,000 Americans age 65 and older [21]. Without knowledge about individual ADLs it will be difficult to improve the success of interventions on ADL difficulty or dependence. Reporting impacts on means of ADL indices and scales is not sufficient, since the same mean can consist of many different combinations of ADL impairments. For instance, a mean of 3 ADLs can consist of bathing, dressing, and eating. Or it could be toileting, transferring, and walking. Or it could be one of many other combinations. Even if a reduction in the mean number of ADLs is achieved, the ADL mix is unknown and will vary from individual to individual. Little progress will be achieved if research continues to utilize means rather than examining effects on each specific ADL.

The present study investigates impacts on difficulty and dependence in individual ADLs, using data from the Medicare Primary and Consumer-Directed Care (PCDC) Demonstration. Medicare is the national program in the United States that finances healthcare for adults age 65 and older, permanently disabled persons under age 65, and individuals with end stage renal disease. Medicare demonstration projects are innovative programs sponsored by Medicare to test the feasibility and effects of new methods of service delivery and payment, and coverage of new services. The Medicare PCDC Demonstration’s HVN intervention had a significant effect on the number of ADLs in which the study subject was dependent [22]. The mean number of ADLs in which study subjects were dependent was lower for the HVN intervention group than for the control group 22 months after study entry. The study that reported this result did not examine HVN impact on ADL difficulty. Subsequent to its publication we carried out both qualitative [23] and quantitative [24] analyses to gain better understanding of how the HVN intervention affected the mean number of ADLs in which individuals were dependent and, separately, the mean number of ADLs they reported having difficulty performing. The present study follows up on our three previous studies by exploring which *individual* ADLs were affected by the Medicare PCDC HVN intervention over 22 months. While the Demonstration had hypotheses for the mean number of ADLs in which patients were dependent or for which they reported difficulty, there were no hypotheses for individual ADLs. Furthermore, we did not anticipate that the HVN intervention would affect every individual ADL for several reasons, including the intervention itself, the nurses, and the patients and their informal caregivers. These reasons are discussed in more detail in the Discussion section. The findings of the exploratory study reported here will be important in beginning to create the knowledge base for future HVN studies on individual ADLs as well as in generating hypotheses for those studies.

## Methods

### Study design

The present study is a secondary analysis of data from the Medicare PCDC Demonstration. It is intended to be the first step of a long-term research process whose ultimate aim will be to determine the mechanisms that explain how HVN interventions affect individual ADLs. The Medicare PCDC Demonstration (1994–2003 with enrolled Medicare patients during 1998–2002) was a multicenter, stratified (by site), unblinded, randomized, controlled, 4-arm parallel-group (1:1:1:1 balanced randomization) study conducted at 2 sites in the United States that enrolled 1,605 Medicare patients [22]. The unit of randomization was the Medicare patient. After trial commencement there were no important changes in methods. The Demonstration was approved by the Centers for Medicare and Medicaid Services (CMS) and the University of Rochester Research Subjects Review Board. Written informed consent for participation in the Demonstration was obtained from all study subjects or a proxy. When the participant was too cognitively impaired to provide written consent, the patient’s primary informal caregiver did so.

### Study setting and participants

The New York State site included 8 counties while 11 counties comprised the West Virginia/Ohio site. A total of 307 primary care physicians was recruited: 249 in New York State and 58 in West Virginia/Ohio. A letter that encouraged enrollment in the study and an application form were mailed to at least some of each physician’s Medicare patients. Eligibility criteria included (1) needing or receiving help with 2+ ADLs or 3+ instrumental ADLs; (2) recent significant healthcare use (hospital inpatient, nursing home, or skilled home healthcare during the past year, or 2+ emergency department visits in the previous 6 months); (3) living in the community; and (4) enrollment in Medicare Parts A and B. Medicare Part A pays for hospital inpatient care, rehabilitation care in a nursing home after discharge from the hospital, home healthcare after hospitalization, and hospice care. Part B finances physician services, hospital outpatient care, emergency department visits, outpatient rehabilitation, renal dialysis, radiation treatment, durable medical equipment, limited home healthcare not following a hospital or nursing home stay, and an array of preventive and screening services.

### Intervention groups

The Demonstration had 4 arms: 3 intervention groups and a control (care as usual) group. The 3 interventions were the HVN intervention, a consumer-directed voucher, and the HVN intervention plus the voucher (combination group). The voucher paid up to $200 per person per month for home and community-based goods and services not normally reimbursed by Medicare including personal care services and companions, environmental modifications, and some types of adaptive and assistive equipment. Since the aim of the present study was to examine the effect on individual ADLs of the HVN intervention only, the present study included 2 groups: the HVN intervention and control groups. The voucher only group was not analyzed here because the intervention its subjects received was not an HVN intervention. The combination group was not analyzed because its subjects received the voucher intervention in addition to the HVN intervention. It is likely that the voucher would have affected ADLs both by itself and in combination with the HVN intervention. We were interested in investigating the effect of the HVN intervention alone. Thus, the other 2 interventions are not described here.

### HVN intervention

The HVN intervention was a disease management-health promotion nurse intervention that included nurse home visiting and was affiliated with primary care. The nurse role focused on empowering patients and educating them about use of behavior change models to facilitate chronic disease self-management. “Hands-on” nursing care was minimal. While an intervention protocol was used to standardize intervention components, a key feature of the intervention was the nurses’ individualization and tailoring of delivery components to meet individual patient needs. ADLs were one of many outcomes at which the intervention was aimed. Others included reducing hospital use, lowering Medicare expenditures, and minimizing custodial nursing home admissions. An unknown proportion of the home visits attempted to specifically address ADL functioning.

Eleven nurses (7 in New York and 4 in West Virginia/Ohio) provided services to 802 patients in both the HVN and combination groups. Prior to the intervention, all nurses received extensive training in aging, geriatric nursing, medication management, health behavior change, and similar topics from a wide array of experienced community and university healthcare professionals.

During the initial home visit the nurse assessed the patient and collected and reviewed the patient’s medications. Following the initial home visit, additional home visits occurred an average of once per month and were approximately one hour long. During these visits the patient’s medications were reviewed and the PRECEDE-PROCEED health education planning model [25] was employed to organize disease prevention, health promotion, chronic disease self-management, and health behavior change and maintenance activities. This framework has been used effectively with older persons to improve self-care management and promote public health. There was often telephone follow-up after the home visit. Positive effects on patient health and disability status, including ADLs, were anticipated because of empirical evidence as well as the theoretical literature [26-28]. With guidance and support from the nurses, patients used two handbooks to help them identify and carry out acute and chronic illness symptom self-care and chronic disease self-management: *Healthwise for Life*[29] and *Consumer Self-Care Strategies* (developed for the Demonstration). These handbooks included physical activity interventions such as exercise for hypertension and diabetes. Information in an exercise manual from the American College of Sports Medicine [30] and a report from the U.S. Surgeon General [31] influenced us to include physical activity in many HVN intervention components. These publications included physical activity interventions for individuals who were disabled due to specific chronic diseases. The nurses were trained and certified as fitness specialists by the Cooper Clinic (Dallas, TX). Physician-patient-family-nurse conferences reimbursed by Medicare ($60 per conference to the primary care physician for up to 4 conferences) were included in the HVN intervention in order to facilitate communication. They were also used to develop plans for disease self-management, carry out ongoing chronic care management, and help resolve emerging conditions. Finally, although the nurses assessed vital signs and body systems, they did not provide most typical “hands-on” nursing care, such as dressing changes, unless participants were at high risk (e.g., had an acute exacerbation of an illness or an emergent need). The HVN intervention is described in detail elsewhere [23,24].

Nurses carried out interventional strategies that aimed at improving or maintaining patients’ performance of specific individual ADLs. These strategies were tailored to each patient’s abilities (see Additional file 1: Table S1).

### Care as usual (Control Group)

The Control group was eligible to receive usual care of all types (hospital, nursing home, home care, and ambulatory) as reimbursed by third parties or self-pay. These included home visits as usually provided by Medicare, other third party payers, and self-pay.

### Data

Data were obtained from several sources. First, data on ADLs and other beneficiary characteristics (demographics, health status, lifestyle, and health insurance) were obtained from a baseline interview of the study participants or their primary informal caregivers administered in the participants’ homes by trained interviewers. Second, follow-up data on ADLs were collected via a 22-month interview administered in the patients’ homes by the interviewers. Third, data on hospital and inpatient rehabilitation expenditures were obtained from the providers after initially being identified in a Health Services Journal completed by each participant or his/her primary informal caregiver on a prospective basis. These were completed from the day patients entered the Demonstration’s intervention phase until they completed or left the Demonstration. Fourth, data on daily use of 9 other healthcare services were obtained from the Health Services Journal. A sample of the utilization data for each service was verified with the providers. Utilization patterns for all services were audited to identify and correct inconsistencies.

### Outcome measures

The outcome measures were data on difficulty performing and dependence in each of 6 individual ADLs at 22 months postbaseline: bathing, dressing, eating, toileting, transferring (getting in or out of bed or chairs), and walking. For each ADL, the 22-month variable was used as the dependent variable in regression models (see below).

ADL dependence [32]: The 6 questions on ADL dependence were: “Tell me how you (the patient) walk and get around/get from bed to chair/get dressed/eat/bathe/use the toilet or commode during the day.” Bathing and dressing dependence had 4 responses, eating and toileting had 5, and transferring and walking 6. For example, the responses for dressing dependence were: 0 = able to dress self without assistance, 1 = able to dress self if clothing is laid out or handed to patient, 2 = able to dress self with some human assistance or supervision, and 3 = depends entirely on another person to dress.

ADL difficulty [33]: These 6 questions asked how difficult it would be for the patient to perform each ADL. The responses were: 0 = no difficulty, 1 = difficulty, and 2 = unable to do.

### Independent variable of interest

The independent variable of interest in each of our regression models was HVN intervention (1 = HVN group and 0 = control group).

### Covariates

We identified 50 potential risk or protective factors for functional disability from those identified in the literature [34,35]. Because of sample size considerations (that is, to meet the rule of thumb for logistic regression that there be at least 15 observations [in this case patients] for each covariate) we then selected 32 covariates for each individual ADL from these 50 factors. They were categorized into the following 6 groups:

• *ADLs/IADLs.—*Baseline difficulty/dependence for the studied ADL, number of ADL difficulties/dependencies at baseline except for the studied ADL, number of IADL difficulties/dependencies, incidence of ADL difficulties/dependencies over the next 22 months except for the studied difficulty/dependency, and incidence of IADL difficulties/dependencies (the incidence variables are counts);

• *Demographics.—*Site, age, female gender, white race, annual household income less than $10,000, married, and high school education or more (except for age these are yes/no binary variables);

• *Health status.—*13 individual chronic conditions, hearing, vision (the preceding are yes/no binary variables), the SF-36 general health perception, mental health, and pain scales (each scored as 0–100), the Cognitive Performance Scale [36], incidence of number of chronic conditions over 22 months, and number of falls in prior month (the last two variables are counts);

• *Lifestyle.—*Number of days per week of physical activity, and lives alone (a yes/no binary variable);

• *Health insurance.—*Medicare supplemental insurance (these are private insurance policies that cover gaps in Medicare such as deductibles and coinsurance), Medicaid (the joint Federal-state government insurance program in the US for persons with low income and assets), and health maintenance organization (these are joint health insurance-delivery organizations which provide a defined set of healthcare services for a monthly payment from a person enrolled in the organization, Medicare, Medicaid, or, for retirees, their former employer) (these are yes/no binary variables); and

• *Health services utilization.—*For the time period the patient was in the study’s intervention phase, the number of nurse home visits, therapist home visits, social worker home visits, home health aide visits, personal care aide hours, companion hours, skilled nursing facility days, custodial nursing home days, and therapist outpatient visits (these are counts), and acute hospital inpatient dollars and inpatient rehabilitation hospital dollars (these are continuous variables).

The same set of covariates was used for the regression models for each ADL (see Additional file 2: Table S2).

### Analyses

For descriptive characteristics, comparisons between the HVN and control groups were performed using t tests for continuous variables and chi-square tests for categorical variables. Comorbidity and the CPS were evaluated using Mann–Whitney U tests.

Three categories of analyses were used to analyze the effects of the HVN intervention on the individual ADLs at 22 months: (1) the traditional approach of unadjusted analyses that strictly depend on random assignment, (2) multinomial logistic regression analyses that adjust for baseline risk factors (adjusted analyses including baseline risk factors can be more efficient than unadjusted analyses and also address any imbalance between the groups that may occur), and (3) multinomial logistic regression analyses that add variables for healthcare services use after randomization (e.g., hospitalization and therapist use) to the baseline risk factors. The third approach is a distinct strength of the study since it adjusts for services the patient received after randomization that are likely to have affected ADLs. We expected that the intervention would reduce the use of some of these services (e.g., hospital and custodial nursing home use) but would increase the use of others (e.g., therapist home visits and personal care aide hours). For other services it was not clear to us what would occur. For example, there could have been less post-acute inpatient rehabilitation hospital use if the intervention kept people healthier and out of the hospital but more use if the nurses convinced patients of the benefit of that service when they were hospitalized. Further, we expected that the mix of services would differ between the intervention and control groups. By including in the model services that could affect ADLs we could see how they differed from the results that just included the intervention and the other covariates in the model. When only the variable for HVN/control group and the other non-use covariates are included, the results are more of a “black box” compared to those that also control for services that will likely affect ADLs. Finally, in the multinomial logistic regression models the dependent variable for each ADL difficulty question had 3 choices while the response for each ADL dependence question had 4–6 choices.

We also estimated binary and ordinal logistic regression models but do not report these results. For ADL *dependence* we report multinomial logistic regression results in part because the Brant test for ordinal logistic regression could not be computed for walking, eating, and transferring dependence. Thus, the ordinal logistic regression results for these three ADLs would be questionable. For bathing, dressing, and toileting dependence the Brant test was satisfactory. For the first two of these ADLs there was little difference between the multinomial and ordinal regression results. For toileting dependence we believe that it is preferable to report the results for the individual dependence categories rather than treat them as ordinal. For ADL *difficulty* we report multinomial rather than ordinal logistic regression because we wanted to be consistent with the dependence results. Moreover, for difficulty the results were essentially the same whether using multinomial or ordinal logistic regression.

All data analyses were performed using STATA version 11.

## Results

### Recruitment

Medicare patients were recruited from May 1997 through June 2000. The study period, which began in July 1998, lasted for 24 months after each participant entered the treatment phase or until he/she died, withdrew, or was disenrolled. The last participant finished in June 2002.

### Participant flow

Application screens were received from 19,469 Medicare patients of the 307 primary care physicians who participated in the Demonstration. Of these 19,469 applicants, 14,978 (76.9%) were excluded because they did not meet Demonstration eligibility criteria. Of the remaining 4,491 applicants, 2,212 (49.2%) were no longer interested, did not meet the technical eligibility criteria, had died, did not live in the community, could not enroll because the enrollment period ended, or were excluded for other reasons. A total of 2,279 of the 4,491 (50.7%) received a baseline interview by research staff. Of these, 493 (21.6%) were unable to enter the Demonstration because they were no longer interested, had died, the approved sample size of 1,600 had been reached, or for several other reasons (see Additional file 3: Figure S1).

### Baseline data numbers analyzed

Of the 766 patients in the HVN intervention (n = 382) and control (n = 384) groups that entered the Demonstration treatment phase, 499 (65.1%) provided information on ADL status at 22 months.

### Patient retention and dropout

One-third (34.8% or 267) of the 766 persons who entered the Demonstration phase are not included in the present study, 139 (18.1%) because they had died, 73 (9.5%) because they met previously defined disenrollment criteria, and 59 (7.7%) because they had voluntarily dropped out. The most serious potential threat to internal validity is differential dropout between the HVN (38.0%; n = 145) and control (31.8%; n = 122) groups. Although HVN patients were more likely to voluntarily drop out (9.7% vs. 4.7%), there was little attrition difference due to death (18.3% vs. 18.0%) or involuntary disenrollment (10.7% vs. 9.4%).

### Baseline patient characteristics

The mean age of the 499 patients in the present study was 76.8 years (standard deviation [SD] = 11.7) (range: 23–103), with 26.4% (n = 132) age 85 and older and 11.8% (n = 59) under age 65 (these individuals were enrolled in Medicare because they were receiving Social Security Disability Insurance benefits, that is, they had been determined by the Federal government’s Social Security program to be permanently disabled). Seven in ten (69.9%) were females. Substantial chronic illness burden (mean number of chronic conditions = 4.3 [SD = 2.2]) and ADL impairment (mean number of ADLs for which difficulty was indicated = 2.74 [SD = 1.82] as well as mean number of ADLs in which the patient was dependent = 1.76 [SD = 1.75]) were reported.

Considerable proportions of the 499 patients had difficulty with or dependence in ADLs. About three-quarters had difficulty with walking (78.0%), about half with transferring (58.5%), bathing (51.7%), or dressing (44.5%), one-quarter with toileting (25.6%) and one-sixth with eating (16.0%). The proportion with dependence was lower than that with ADL difficulty but still substantial for walking (58.7%), transferring (36.3%), bathing (32.3%), and dressing (25.4%). The proportion with eating dependence (15.8%) was essentially the same as for eating difficulty, while toileting dependence was lowest (8.0%). Sociodemographic, ADL, health status, lifestyle, and health insurance characteristics of the HVN intervention (n = 237) and control (n = 262) groups were quite similar (see Table 1).

**Table 1:** Baseline patient characteristics

	**All patients (N = 766)**	**Patients analyzed**			**Patients not analyzed (n = 267)**
		**HVN and control groups (n = 499)**	**HVN group (n = 237)**	**Control group (n = 262)**	
**Sociodemographics**					
Age, mean (SD)	77.7 (11.4)	76.8 (11.8)	77.0 (10.9)	76.7 (12.5)	79.3 (10.7)
Female, n (%)	527 (68.8)	349 (69.9)	166 (70.0)	183 (69.8)	178 (66.7)
Nonwhite, n (%)	25 (3.3)	14 (2.8)	6 (2.5)	8 (3.0)	11 (4.1)
Annual household income					
≤ $10,000, n (%)	255 (33.3)	170 (34.1)	83 (35.0)	87 (33.2)	85 (31.8)
$10,000-$19,999, n (%)	261 (34.1)	166 (33.3)	76 (32.1)	90 (34.4)	95 (35.6)
$20,000-$29,999, n (%)	141 (18.4)	90 (18.0)	45 (19.0)	45 (17.2)	51 (19.1)
$30,000+	109 (14.2)	73 (14.6)	33 (13.9)	40 (15.3)	36 (13.5)
Married, n (%)	316 (41.2)	213 (42.7)	96 (40.5)	117 (44.7)	103 (38.6)
No high school diploma, n (%)	292 (38.1)	185 (37.1)	96 (40.5)	89 (34.0)	107 (40.1)
New York site, n (%)	515 (67.2)	330 (66.1)	156 (65.8)	174 (66.4)	185 (69.3)
**Activities of daily living**					
**Any difficulty**					
Bathing	434 (56.6)	258 (51.7)	122 (51.5)	136 (51.9)	176 (65.9)
Dressing	380 (49.6)	222 (44.5)	105 (44.3)	117 (44.7)	158 (59.2)
Eating	151 (19.7)	80 (16.0)	45 (19.0)	35 (13.4)	71 (26.6)
Toileting	229 (29.9)	128 (25.6)	65 (27.4)	63 (24.0)	101 (37.8)
Transferring	462 (60.3)	292 (58.5)	145 (61.2)	147 (56.1)	170 (63.7)
Walking	608 (79.4)	389 (78.0)	186 (78.5)	203 (77.5)	219 (82.0)
**Any dependence**					
Bathing	301 (39.3)	161 (32.3)	76 (32.1)	85 (32.4)	140 (52.4)
Dressing	237 (30.9)	127 (25.4)	57 (24.0)	70 (26.7)	110 (41.2)
Eating	163 (21.3)	79 (15.8)	37 (15.6)	42 (16.0)	84 (31.5)
Toileting	92 (12.0)	40 (8.0)	16 (6.8)	24 (9.2)	52 (19.5)
Transferring	297 (38.8)	181 (36.3)	75 (31.6)	106 (40.5)	116 (43.4)
Walking	475 (62.0)	293 (58.7)	138 (58.2)	155 (59.2)	182 (68.2)
**Health status**					
Number of chronic conditions, mean (SD)	4.4 (2.2)	4.3 (2.2)	4.5 (2.4)	4.2 (2.1)	4.4 (2.1)
Cognitive Performance Scale score, mean (SD)	1.2 (1.4)	1.0 (1.2)	1.0 (1.1)	1.1 (1.2)	1.5 (1.6)
SF-36 domains					
General health perceptions, mean (SD)	44.8 (21.3)	45.8 (21.4)	44.2 (20.7)	47.3 (21.9)	43.0 (21.0)
Mental health index (MHI-5), mean (SD)	61.9 (16.4)	61.6 (16.5)	62.0 (15.2)	61.2 (17.5)	62.6 (16.4)
Pain, mean (SD)	50.7 (21.0)	51.2 (27.0)	51.0 (27.1)	51.3 (26.8)	49.7 (27.2)
**Lifestyle**					
Physical activity days per week, mean (SD)	3.9 (3.2)	4.1 (3.1)	4.3 (3.0)	4.0 (3.1)	3.4 (3.3)
Lives alone	280 (36.6)	182 (36.5)	93 (39.2)	89 (34.0)	98 (36.7)
**Health insurance**					
Medicare supplemental insurance	545 (71.2)	353 (70.7)	168 (70.9)	185 (70.6)	192 (71.9)
Medicaid	85 (11.1)	53 (10.6)	22 (9.3)	31 (11.8)	32 (12.0)
Health maintenance organization	82 (10.7)	53 (10.6)	22 (9.3)	31 (11.8)	29 (10.9)

### ADL differences at baseline

**All participants** One ADL, eating, differed significantly in prevalence of any difficulty between the HVN (23.8%) and control (15.6%) groups (p = .01) whereas a second ADL, transferring, differed significantly in dependence (HVN group = 43.2% versus control group = 34.3%: p = .01).

**Participants with 22-month data** One ADL, transferring, differed significantly in dependence prevalence between the HVN (31.6%) and control (40.4%) groups (p = .04).

### ADL comparisons at 22 months

**ADL Difficulty** In unadjusted analyses for categories of ADL difficulty (no, some, or great difficulty), a higher proportion of HVN patients had no bathing difficulty (p ≤ .05) in comparison to the control group. In multinomial regression models adjusting for baseline risk factors, the HVN group was significantly less likely to experience some difficulty (OR = 0.58; p ≤ .05) and great difficulty (OR = 0.40; p ≤ .01) for bathing, and great difficulty (OR = 0.39; p ≤ .01) for dressing as compared with the control group. When covariates adjusting for health services received after study entry were added to the risk factors, the proportion of individuals in the HVN group reporting some difficulty (OR = 0.47; p ≤ .01) and great difficulty (OR = 0.38; p ≤ .05) for bathing remained significantly lower.

**ADL Dependence** In unadjusted analyses at 22 months, a higher proportion of HVN patients was independent in toileting (p ≤ .05). In multinomial regression models adjusting for baseline risk factors, the proportion of patients having some human assistance for toileting was significantly lower (OR = 0.51; p ≤ .05) for the HVN group. When covariates adjusting for healthcare services after study entry were added, fewer patients were totally dependent in toileting (OR < 0.01; p ≤ .01) and more patients used a bedpan or urinal (OR > 10.00; p ≤ .01). Also, in the HVN group more patients were fed entirely by tube or other means (OR = 79.0; p ≤ .01) while fewer were bedfast (OR < 0.01; p ≤ .01), including being bedfast and able to turn in bed (OR = 0.08; p ≤ .01) and being bedfast and unable to turn (OR < 0.01; p ≤ .01) (see Table 2).

**Table 2:** Categories of difficulty or dependence for individual ADLs (multinomial analysis): unadjusted absolute percent difference and adjusted odds ratio (OR) of the Home Visiting Nurse Intervention Group as compared to the Control Group at 22 Months

**ADL**	**Difficulty**				**Dependence**			
		**Unadjusted difference**	**Adjusted OR (95% CI)**			**Unadjusted difference**	**Adjusted OR (95% CI)**	
			**Model 1**	**Model 2**			**Model 1**	**Model 2**
Bathing	No difficulty	+9.7**	Reference	Reference	Independent	+3.0	Reference	Reference
	Some difficulty	-4.0	0.58** (.37-.90)	0.47*** (.27-.81)	Some human assistance	-0.4	0.86 (.51-1.46)	1.08 (.58-.2.03)
	Great difficulty	-5.7*	0.40*** (.20-.81)	0.37** (.16-.85)	Requires another person	-1.3	0.76 (.42-1.37)	1.36 (.62-2.99)
					Totally by another person	-2.8	0.49 (.18-1.30)	1.13 (.30-4.28)
Dressing	No difficulty	+5.9	Reference	Reference	Independent	+4.9	Reference	Reference
	Some difficulty	-1.1	0.75 (.48-1.17)	0.81 (.48-1.38)	Clothing laid/handed out	-1.4	0.72 (.30-1.74)	1.04 (.37-2.94)
	Great difficulty	-4.8*	0.39*** (.18-.82)	0.67 (.26-1.75)	Some human assistance	-0.2	0.82 (.48-1.39)	1.03 (.48-2.21)
					Entirely dependent	-3.3	0.68 (.29-1.62)	1.14 (.36-3.62)
Eating	No difficulty	+2.0	Reference	Reference	Independent	+1.6	Reference	Reference
	Some difficulty	-0.3	0.84 (.50-1.43)	0.98 (.55-1.73)	Intermittent assistance	-1.7	0.90 (.50-1.61)	1.23 (.63-2.40)
	Great difficulty	-1.8	0.36 (.10-1.26)	1.10 (.08-14.69)	Assisted throughout meal	-1.6	0.62 (.18-2.13)	1.40 (.30-6.48)
					Supplemented by tube	+0.4		2.14* (.96-4.78)
					Fed entirely by tube/other	+1.3	2.39 (.72-7.89)	79.0*** (22.1-282.5)
Toileting	No difficulty	+7.4*	Reference	Reference	Independent	+6.6**	Reference	Reference
	Some difficulty	-6.8	0.70 (.44-1.12)	0.73 (.42-1.29)	Some human assistance	-4.2	0.51** (.26-1.01)	0.46 (.15-1.43)
	Great difficulty	-0.6	0.76 (.27-2.09)	1.14 (.32-3.99)	Uses bedside commode	+0.3	1.42 (.52-3.86)	0.32 (.07-1.52)
					Uses bedpan or urinal	-0.3	0.34 (.03-3.90)	>10.00*** (7.28-1.2 to 12th power)
					Totally dependent	-2.4	0.63 (.19-2.09)	<0.01*** (5.0 to -9th power -.03)
Transferring	No difficulty	-0.2	Reference	Reference	Independent	+6.3	Reference	Reference
	Some difficulty	+1.8	1.14 (.72-1.79)	1.30 (.76-2.20)	Minimal assistance/device	-5.7	0.89 (.56-1.39)	0.73 (.40-1.31)
	Great difficulty	-1.6	0.82 (.35-1.90)	1.45 (.43-4.88)	Can bear weight when transfer	+0.6	1.03 (.42-2.54)	1.86 (.52-6.64)
					Unable to bear weight	+0.4		0.92 (.04-23.0)
					Bedfast – able to turn	-0.4		0.08*** (.03-.22)
					Bedfast – unable to turn	-1.2*		<0.01*** (<.01- < .01)
					Unable to bear weight/bedfast		0.98 (.30-3.18)	
Walking	No difficulty	+4.6	Reference	Reference	Independent	+6.0	Reference	Reference
	Some difficulty	-2.9	0.90 (.53-1.54)	0.79 (.42-1.47)	Some human assistance	-3.3		
	Great difficulty	-1.7	0.76 (.34-1.69)	0.68 (.25-1.84)	Device/human assistance		0.78 (.47-1.30)	0.71 (.41-1.23)
					Walk only with assistance	-1.2	0.44 (.13-1.56)	0.56 (.14-2.16)
					Wheels self independently	+1.6		
					Chairfast – wheels self		0.83 (.32-2.12)	1.86 (.48-7.19)
					Chairfast	-1.6		
					Chairfast – cannot wheel self			0.24* (.06-1.08)
					Chairfast – cannot wheel self – or bedfast		0.37 (.10-1.42)	
					Bedfast	-1.5*		<0.01*** (<.01- < .01)

^*^p ≤ .10; ^**^p ≤ .05; ^***^p ≤ .01.

Note: CI = Confidence Interval. Unadjusted differences are absolute (not relative) differences. A minus sign means that the intervention group has a smaller proportion with difficulty or dependence than the control group. Model 1 adjusts for demographics, health status, lifestyle, and health insurance. Model 2 adds healthcare services use after baseline to the variables included in Model 1.

## Discussion

With two exceptions, a home visiting nurse intervention with monthly home visits whose goal was to empower Medicare patients to successfully carry out chronic disease self-management appeared to have little effect on individual ADLs, measured as either difficulty or dependence, compared to care as usual 22 months after the beginning of the intervention. The exceptions are (1) less difficulty performing bathing, and (2) effects for the most dependent levels of four ADLs: less dependence in walking and transferring, a substitution effect for toileting, and more dependence in eating. An important caveat is that there were only a small number of patients, a few dozen, in the most dependent levels of the latter four ADLs.

The most logical explanation for the finding of less bathing difficulty is the services provided by the HVN intervention itself. The HVN nurses worked with patients and caregivers to help patients gain mastery of bathing sub-tasks, assisted them in modifying the home environment through the acquisition and use of adaptive equipment, and devised strategies to support participants in dealing with fatigue/pain that could prevent independent bathing. There is growing evidence that the type of bathing intervention used by the HVN intervention, one individualized to the preferences and goals of older persons, is more effective in forestalling disability [37].

Why did the HVN intervention appear to affect bathing difficulty but not difficulty for the other five ADLs? One possible reason is that it is easier to reduce bathing difficulty than it is to reduce difficulty performing other ADLs when individuals are experiencing difficulty with bathing and other ADLs. Many studies have found that bathing is the first ADL with which people experience difficulty [7,38-40], including the finding that bathing tends to enter first among 103 ADL hierarchies that meet the minimum requirements for scalability [41]. Moreover, older persons are more likely to have negative expectations regarding the inevitability of further physical decline after they experience bathing difficulties [42]. As older persons adapt to decline they may be more likely to accept personal assistance for bathing. This may have the unintended consequence of engendering reliance on this assistance, in turn leading to more functional decline—in performance of other ADLs. Thus, the presence of bath aids or help may be useful in managing bathing but may not be helpful in preventing decline in other ADLs [43]. Nurses should try to decrease patients’ negative expectations about the inevitable decline of their other ADL abilities at the same time as they implement strategies to promote independent bathing.

Impacts relating to dependence were identified for the most dependent levels of four ADLs – walking, transferring, toileting, and eating – but not for any levels of dependence for bathing and dressing, and not for lower or middle levels of dependence for walking, transferring, toileting, and eating. An important caveat of this finding is that very few patients, a few dozen, were at the most dependent levels for these four ADLs.

**Walking and transferring dependence** Compared to the control group, fewer patients were bedfast at 22 months, including both patients who were able and unable to turn themselves in bed. The intervention may have empowered the most disabled patients to focus on specific tasks aligned with their needs (e.g., rolling out of bed) rather than on generic tasks (e.g., increasing range of motion and ambulation targeted to maintaining activity and flexibility).

**Toileting dependence** A substitution effect appears to have occurred for toileting dependence, with fewer patients totally dependent in toileting at 22 months. Nurses intervening to help participants and caregivers identify or implement simple modifications to address environmental barriers (e.g., using a bedpan or urinal) may account for more patient independence in self-toileting.

**Eating dependence** An unexpected result was greater dependence in eating. More patients were fed entirely by tube feeding or other means. Perhaps we should not have been surprised by this finding because the intervention focused on addressing nutritional risk factors rather than on promoting independence in eating. Also, the nurse may have recognized a nutritional deficit when the patient was dependent in eating and suggested tube feeding or nutritional supplements.

Another interpretation of our findings stresses the presence of no effects for most ADLs more and the effects on bathing difficulty and the most dependent levels of four other ADLs less. This interpretation is that, overall, the HVN intervention had little impact on individual ADLs. There are several plausible reasons for this interpretation.

First, the HVN intervention simply may not have “worked”. This could have been due to its design or its implementation or both. Its design may have been such that the intervention could not have reasonably been expected to affect most individual ADLs. While it was expected that the patients in the HVN group would have had better outcomes for ADLs than the control group would have had, ADLs were only one of many demonstration goals and hence may not have been given sufficient attention in study design or implementation. Furthermore, our expectation that individual ADLs would be directly affected by improvement in goals specifically related to individual ADLs as well as indirectly through improved medication management, physical activity, diet, and empowerment may not have been correct.

Second, the nurses may not have done what was needed to achieve less difficulty or more independence for individual ADLs as compared to care as usual. There are several ways this could have occurred. (a) The nurses could have done everything they were expected to do under demonstration policies and procedures, but what was called for in these policies and procedures may simply have been insufficient to achieve better individual ADL outcomes. (b) The nurses may not have sufficiently followed policies and procedures, although we do not believe that this was the case because they were carefully supervised. (c) Competing demands [44] on the nurses could have resulted in the intervention focusing on diseases, conditions, activities, and tasks other than ADLs. Related to this is that the nurses had additional tasks to perform when patients experienced acute illnesses or had chronic disease exacerbations, and made visits to hospitalized patients and patients receiving post-hospital rehabilitation in nursing homes and rehabilitation facilities.

Third, because the study patients were quite sick and disabled, with many on a downward trajectory, achieving better individual ADL outcomes compared to the control group may not have been possible. Too few patients may have had ADL goals, and meeting those goals may have been insufficient to achieve better ADL outcomes in comparison to care as usual. Furthermore, patients and their informal caregivers may not have been sufficiently motivated to work on their ADL goals or on other activities, such as medication management that could have been expected to affect ADLs. It was difficult for patients and caregivers to work on ADL goals and other activities when interrupted by serious acute illnesses and chronic disease exacerbations, including those that resulted in hospitalizations. Another possible reason for the absence of effects on most individual ADLs is that competing demands on the patient and/or primary informal caregiver resulted in the intervention focusing on diseases, conditions, activities, and tasks other than ADLs. The intervention’s focus on patient empowerment may have led some patients to choose to spend their time and energy on other activities such as improving medication adherence or learning about chronic illnesses rather than on ADLs. For some patients the presence of dominant comorbid conditions (e.g., stage IV heart failure) or significant socio-economic, cultural, or environmental barriers might have precluded the addressing of ADLs.

Fourth, the ADL measures themselves are problematic. They are ordinal scores rather than interval level measures [45], and have from three to 6 levels. While they were included in CMS’s OASIS instrument and in CMS’s Health of Seniors survey, they may not have been able to detect differences between the HVN and control groups even when such differences were present.

### Limitations

Several limitations must be acknowledged. First, the drop from 766 patients in the HVN intervention and control groups at baseline to 499 in the analysis at 22 months raises questions about selection bias and validity of the results. However, the 499 patients whose data were analyzed at 22 months were not appreciably different at baseline from the entire group of 766 patients in terms of any ADL difficulty (an average of 3.4% less likely across all 6 ADLs) or ADL dependence (a mean of 4.6% less likely for all 6 ADLs). Furthermore, the large number of variables that control for differences between the HVN and control groups should greatly lessen any selection bias that might have occurred. Second, the HVN group experienced a higher dropout rate during the intervention period than the control group, 38.0% versus 31.8%. This differential attrition was due to voluntarily drop-out (9.7% vs. 4.7%) rather than to death or involuntary disenrollment. It may have been due to individuals in the HVN group not wanting to carry out various chronic disease self-management activities. Third, it would have been desirable to examine the HVN effect for those patients who had identified ADL improvement/maintenance as a goal for specific ADLs. However, limiting the analysis to the small proportion of patients with this goal would ignore the indirect effects of the intervention on all patients’ ADLs through other aspects of the intervention such as medication management, greater physical activity, improved diet, and patient empowerment that are likely to affect ADLs. Fourth, generalizability may be limited because the participants were from 19 counties in 3 states in one nation and the sample was predominantly white. There were limited numbers of older minorities in the Demonstration counties. Fifth, the data used in this study are dated, with the last patient completing the treatment phase in 2002. However, little has changed since then in terms of HVN interventions in the US providing services to non-dual eligible older adults living at home over long periods of time. Dual eligibles are individuals enrolled in both Medicare and Medicaid. About 90% of the Demonstration patients were non-dual eligibles. While Medicare home healthcare delivery has changed since 2002, it typically provides services for a much shorter period of time (often 60 days or less) than the 22-month timeframe of our analysis. A study strength is that it examines the HVN effect over such a long timeframe. Sixth, an issue with our study is adequate control over type I error since we are exploring 116 possible effects. Some would use an approach such a Bonferroni correction or simply reduce the p value from the customary value of .05 to .01 or another value. However, in a study such as ours that is examining an issue for the first time, we believe that it is better to use the conventional value of .05 and err on the side of falsely accepting the test. Seventh, our “non-findings” are null findings that cannot be proven.

### Clinical practice, public policy, and research implications

It would be premature to develop clinical practice or public policy implications or recommendations from the results of this single study until additional research is carried out. Importantly, however, our study does have implications for future research. The most obvious research implication is to evaluate the data sets for the previous HVN studies that found positive effects on the mean number of ADL indices or scales but did not examine effects for individual ADLs [8-18,20]. In those studies, data on individual ADLs were collected and calculated to determine the effects on the mean number of indices or scales. While some of these studies are dated, it would be worthwhile to analyze those that are recent. Second, it is possible that some of the studies that failed to detect significant effects on mean numbers or indices of ADLs were effective for some individual ADLs, the mean non-effect being a “canceling out” of positive and negative effects. Data sets for those studies should be assessed as well. The evaluation of both previous successful and unsuccessful HVN studies will help the field to reach the next level of understanding relatively quickly since those data are already in existence. Third, qualitative investigation is required to better understand the perspectives of older persons relating to individual ADLs to better inform the development of effective patient-centered interventions. Finally, in previous research we found that maintaining/improving the number of ADLs was associated with two structure components of our HVN intervention – education materials and special physician-patient-family-nurse conferences – and three process components – goal setting, disease management, and medication management [24]. It is currently unknown which structure and process components of HVN interventions affect which individual ADLs. Research is needed to determine this.

## Conclusions

This study found that a home visiting nurse intervention resulted in less difficulty performing bathing but had little effect on difficulty for five other ADLs. Although no differences were found for lower levels of dependence for any ADLs, impacts were present for the most dependent levels of walking, transferring, toileting, and eating. Since little is known about the effects of HVN interventions on individual ADLs, research should be carried out examining specific ADLs in recent existing data sets of previous HVN studies that analyzed the mean number of ADL indices or continuous scales.

## Competing interests

The authors declare that they have no competing interests.

## Authors’ contributions

BF planned the study, supervised the data analysis, and wrote the Introduction, much of the Methods and Results, and most of the Discussion. YL performed all statistical analyses and wrote portions of the Methods and Results. DVL wrote sections of the Methods and Discussion. BAP read the entire manuscript and contributed to its revision. All authors read and approved the final manuscript.

## Pre-publication history

The pre-publication history for this paper can be accessed here:

http://www.biomedcentral.com/1471-2318/14/24/prepub

## Supplementary Material

Additional file 1: Table S1 Nurse intervention activities and tasks. Click here for file

Additional file 2: Table S2 Independent variables selected for regression models. Click here for file

Additional file 3: Figure S1 Flowchart of participants through each stage of the demonstration. Click here for file
