# Supplementary material for: Effects of a home visiting nurse intervention versus care as usual on individual activities of daily living: a secondary analysis of a randomized controlled trial
Source: BMC Geriatr. 2014 Feb 20;14:24. doi: 10.1186/1471-2318-14-24 (PMC3933382; doi:10.1186/1471-2318-14-24)
Supplement: Additional file 1: Table S1 — Nurse intervention activities and tasks. [file 1471-2318-14-24-S1.docx]

Supplementary Table 1: Nurse Intervention Activities and Tasks

| ADL | Nurse Intervention Activities and Tasks |
| --- | --- |
| Bathing | - Helped participants create goals targeted to strategies found effective in previous studies. - Worked with participants and caregivers to gain mastery of the sequential sub-tasks involved in bathing (e.g., getting in and out of the tub). - Helped participants modify the home environment and identify safety hazards (e.g., purchase and teach the use of adaptive equipment). - Devised strategies to help participants deal with fatigue/pain from multiple diseases that prevented independent bathing. |
|  |  |
| Dressing | - Matched the level of participation individuals were physically/-cognitively capable of to self-care management goals and strategies (e.g., used adapted equipment/clothing to accommodate paresis from stroke). - Individualized care management goals by considering predisposing and enabling factors used to structure and personalize care (e.g., considered participants’ established practices and preferences in dressing). - Helped participants relearn dressing techniques or learn new chains of dressing. - Attempted to empower participants and caregivers to obtain in-home occupational therapy or other specialist care. |
| Eating | - Primarily focused on addressing nutritional risk factors rather than on promoting independence in eating. - Prioritized the negative impact of poor nutritional status (e.g., low BMI, malnutrition, and dehydration) over issues associated with eating/feeding. - Attempted to empower participants or caregivers to improve nutrition. |
| Toileting | - Helped participants and caregivers to become more independent in toileting through assessment of urinary continence (e.g., presence/type of urinary incontinence) and symptoms of urinary dysfunction (e.g., nocturia). - Assisted patients and caregivers to create individually tailored treatment goals that included strategies targeted to continence management (e.g., bladder training, and prompted voiding techniques). - Supported patients to communicate effectively with physicians to request pharmacotherapy to treat incontinence as well as identify medications potentially associated with urinary retention/frequency. - Helped participants and caregivers identify adaptations (e.g., hand rails; raised toilet seats) or implement simple modifications to environmental barriers (e.g., commode) that allowed independence in self-toileting. |
| Transferring | - Provided psycho-social support and education related to prevention of complications associated with immobility (e.g., improving skin integrity, hydration/fluid balance). - Attempted to empower caregivers to encourage participant independence by not routinely assisting with transfers (e.g., educating caregiver to help only when necessary). - Helped participants become knowledgeable about safety measures used during transfers (e.g., transfer boards). |
| Walking | - Limited exercise and mobility training (e.g., strength/gait training, tai chi) - Actively engaged participants and caregivers to perform low-intensity. disease-specific aerobic chair exercises to increase flexibility, balance, and walking (e.g., Sit and Be Fit exercises). - Attempted to empower participants to use assistive devices such as cane, walker, or crutches to increase walking independence. - Advocated for appropriate resources or referrals (e.g., attempted to empower participants to ask for physical therapy on physician/nurse/participants visits). - Assessed home environment for factors that created physical barriers to walking (e.g., scatter rugs associated with falls). |

Note: (1) Not all patients received each of these strategies for a given ADL. Rather, strategies were tailored to specific patient needs and wants. (2) Techniques used to assist participants learn techniques included such tasks as modeling the skill or breaking it down into small sequential steps. An example is teaching someone how to get in and out of the bathtub.
