# Supplementary material for: Effects of a home visiting nurse intervention versus care as usual on individual activities of daily living: a secondary analysis of a randomized controlled trial
Source: BMC Geriatr. 2014 Feb 20;14:24. doi: 10.1186/1471-2318-14-24 (PMC3933382; doi:10.1186/1471-2318-14-24)
Supplement: Additional file 2: Table S2 — Independent variables selected for regression models. [file 1471-2318-14-24-S2.docx]

Additional file 2: Table S2. Independent Variables Selected for Regression Models

| Panel A | | | | | | |
| --- | --- | --- | --- | --- | --- | --- |
|  | Dependence | | | | | |
|  | Bathing | Dressing | Walking | Transferring | Toileting | Eating |
| Home visiting intervention | √ | √ | √ | √ | √ | √ |
| Baseline dependence | √ | √ | √ | √ | √ | √ |
| Number of ADL dependencies at baseline except the studied dependency | √ | √ | √ | √ | √ | √ |
| Number of IADL dependencies | √ | √ | √ | √ | √ | √ |
| Incidence of ADL dependencies except the studied dependency | √ | √ | √ | √ | √ | √ |
| Incidence of IADL dependencies | √ | √ | √ | √ | √ | √ |
| Incidence of chronic conditions | √ | √ | √ | √ | √ | √ |
| New York site | √ | √ | √ | √ | √ | √ |
| Age | √ | √ | √ | √ | √ | √ |
| Female gender | √ | √ | √ | √ | √ | √ |
| White | √ | √ | √ | √ | √ | √ |
| Income below $10,000 per year | √ | √ | √ | √ |  | √ |
| Married | √ | √ | √ | √ |  |  |
| High school education | √ | √ | √ |  | √ | √ |
| Number of falls |  | √ | √ | √ | √ | √ |
| SF-36 Mental Health Index score | √ | √ |  |  | √ |  |
| SF-36 General Health Perception Scale score |  | √ | √ |  | √ |  |
| Hearing | √ |  |  |  |  |  |
| Vision | √ | √ |  | √ | √ |  |
| Cognitive Performance Scale score | √ | √ |  | √ | √ | √ |
| SF-36 Pain Scale |  |  | √ | √ | √ | √ |
| Number of chronic conditions | √ |  | √ |  | √ | √ |
| Hip/knee arthritis | √ | √ |  | √ | √ | √ |
| Wrist/hand arthritis |  | √ |  | √ | √ | √ |
| Myocardial infarction |  | √ |  |  |  | √ |
| Chronic obstructive pulmonary disease | √ |  | √ |  | √ | √ |
| Crohn’s disease |  | √ |  |  |  | √ |
| Cancer | √ |  |  |  |  | √ |
| Stroke | √ |  | √ | √ | √ | √ |
| Diabetes |  |  |  | √ |  |  |
| Other heart conditions | √ | √ | √ | √ |  |  |
| Angina |  |  | √ |  |  | √ |
| Chronic heart failure |  |  | √ |  | √ | √ |
| Hypertension |  | √ | √ |  | √ | √ |
| Sciatica | √ |  |  |  |  |  |
| Physical activities | √ |  | √ | √ | √ |  |
| Live alone | √ |  |  |  | √ |  |
| Hours of service provided by home health aides | √ |  |  |  |  |  |
| Home visits by social workers |  |  | √ | √ | √ | √ |
| Home visits by personal care aides | √ | √ |  | √ | √ |  |
| Hours of companionship | √ | √ |  | √ |  | √ |
| Home visits by nurses |  | √ | √ | √ |  | √ |
| Home visits by therapists |  | √ |  | √ |  |  |
| Outpatient visits by therapists |  | √ | √ | √ | √ |  |
| Cost of acute hospital inpatient services (dollars) |  | √ | √ | √ |  |  |
| Cost of inpatient rehabilitation services (dollars) | √ |  | √ | √ | √ | √ |
| Days of skilled nursing facility usage | √ |  |  | √ | √ |  |
| Days of custodial nursing home usage |  | √ | √ | √ | √ | √ |
| Medicare supplemental insurance |  |  |  |  |  |  |
| Medicaid | √ |  | √ |  |  |  |
| Health maintenance organization | | √ | √ |  |  | √ |

| Panel B | | | | | | | |
| --- | --- | --- | --- | --- | --- | --- | --- |
|  | Difficulty | | | | | | |
|  | Bathing | | Dressing | Walking | Transferring | Toileting | Eating |
| Home visiting intervention | √ | | √ | √ | √ | √ | √ |
| Baseline difficulty | √ | | √ | √ | √ | √ | √ |
| Number of ADL difficulties at baseline except the studied difficulty | √ | | √ | √ | √ | √ | √ |
| Number of IADL difficulties | √ | | √ | √ | √ | √ | √ |
| Incidence of ADL difficulties except the studied difficulty | √ | | √ | √ | √ | √ | √ |
| Incidence of IADL difficulties | √ | | √ | √ | √ | √ | √ |
| Incidence of chronic conditions | √ | | √ | √ | √ | √ | √ |
| New York site | √ | | √ | √ | √ | √ | √ |
| Age | √ | | √ | √ | √ | √ | √ |
| Female gender | √ | | √ | √ | √ | √ | √ |
| Caucasian American | √ | | √ | √ | √ | √ | √ |
| Income below $10,000 per year |  | | √ | √ | √ |  | √ |
| Married | √ | | √ | √ | √ | √ | √ |
| High school education | √ | |  |  | √ | √ |  |
| Number of falls |  | |  | √ | √ |  |  |
| Sf-36 Mental Health Index | √ | | √ | √ |  | √ | √ |
| Sf-36 General Health Perception Index | √ | |  |  |  |  | √ |
| Hearing |  | | √ |  | √ |  | √ |
| Vision | √ | |  | √ | √ |  | √ |
| Cognitive Performance Scale score | √ | | √ | √ |  |  | √ |
| Sf-36 Pain Index | √ | |  |  |  | √ | √ |
| Number of chronic conditions | √ | |  | √ |  | √ | √ |
| Hip arthritis |  | | √ | √ |  | √ |  |
| Wrist arthritis | √ | | √ |  |  |  |  |
| Myocardial infarction |  | | √ | √ |  | √ | √ |
| Chronic obstructive pulmonary disease | √ | |  |  | √ | √ | √ |
| Crohn’s disease |  | |  | √ |  |  | √ |
| Cancer |  | |  |  |  |  |  |
| Stroke | √ | | √ | √ | √ |  | √ |
| Diabetes |  | |  | √ | √ |  | √ |
| Other heart conditions |  | | √ |  | √ |  |  |
| Angina | √ | |  |  |  | √ |  |
| Chronic heart failure |  | |  |  |  | √ |  |
| Hypertension |  | |  | √ |  | √ | √ |
| Sciatica pain |  | |  |  |  |  |  |
| Physical activities |  | | √ | √ | √ | √ | √ |
| Live alone | √ | | √ | √ |  | √ | √ |
| Hours of service provided by home health aides | √ | | √ |  |  | √ | √ |
| Home visits by social workers |  | |  | √ | √ |  |  |
| Home visits by home health aides | √ | | √ | √ | √ | √ | √ |
| Hours of companionship | √ | |  | √ | √ |  |  |
| Home visits by nurses | √ | | √ |  | √ |  | √ |
| Home visits by therapists | √ | | √ |  | √ | √ | √ |
| Outpatient visits by therapists |  | | √ | √ | √ | √ |  |
| Cost of acute inpatient services (dollars) | √ | | √ | √ |  | √ |  |
| Cost of inpatient rehabilitation services (dollars) | | |  |  | √ | √ |  |
| Days of skilled nursing home usage | |  | √ | √ | √ | √ |  |
| Days of custodial nursing home usage | |  | √ |  | √ | √ |  |
| Medigap insurance | | √ | √ | √ | √ | √ | √ |
| Medicaid enrollment | | √ |  |  | √ |  |  |
| Health maintenance organization enrollment | | √ | √ |  |  |  |  |
