# Supplementary material for: Effects of a home visiting nurse intervention versus care as usual on individual activities of daily living: a secondary analysis of a randomized controlled trial
Source: BMC Geriatr. 2014 Feb 20;14:24. doi: 10.1186/1471-2318-14-24 (PMC3933382; doi:10.1186/1471-2318-14-24)
Supplement: Additional file 3: Figure S1 — Flowchart of participants through each stage of the demonstration. [file 1471-2318-14-24-S3.docx]

**Figure 1. Flowchart of Participants through Each Stage of the Demonstration**

Excluded (n=493)

Enrollment period ended (n=307)

No longer interested (n=79)

Deceased (n=28)

Does not live in community (n=18)

Receives Medicare Hospice benefit (n=9)

Other (n=52)

Excluded (n=2,212)

No longer interested (n=503)

Deceased (n=363)

Enrollment period ended (n=318)

Does not live in community (n=216)

Medicare TEFRA HMO enrollee (n=152)

Does not have both Medicare A and B (n=51)

Physician dropped out of study (n=51)

Has Medicaid (n=50)

Other (n=508)

4,491 Eligibility verified by HCFA

2,279 Assessment completed

Excluded (n=14,978)

Did not meet program eligibility criteria (n=12,456)

Deceased (n=735)

Medicare TEFRA HMO enrollee (n=636)

No longer interested (n=323)

Does not live in community (n=216)

Has Medicaid (n=154)

Cannot be located (n=89)

Does not have both Medicare A and B (n=77)

Resides outside catchment area (n=74)

Physician refused or withdrew approval (n=69)

Medicare is secondary payer (n=52)

Other (n=97)

19,469 Applications received

Combination Intervention (n=445)

Did not receive intervention (n=25)

Does not live in community (n=8)

Deceased (n=6)

No longer interested (n=4)

Other (n=7)

1,786 Randomized

Completed entire 24 months (n=237)

Entered intervention phase (n=384)

Completed entire 24 months (n=301)

Completed entire 24 months (n=262)

Entered intervention phase (n=420)

Completed entire 24 months (n=285)

Entered intervention phase (n=382)

Entered intervention phase (n=419)

Control Intervention (n=459)

Did not receive intervention (n=75)

No longer interested (n=57)

Deceased (n=5)

Patient cannot complete paperwork (n=4)

Other (n=9)

HVN Intervention (n=443)

Did not receive intervention (n=61)

No longer interested (n=37)

Does not live in community (n=9)

Deceased (n=5)

Cannot complete paperwork (n=3)

Other (n=7)

Voucher Intervention (n=439)

Did not receive intervention (n=20)

No longer interested (n=8)

Deceased (n=2)

Resides outside catchment area (n=2)

Medicaid Waiver (n=2)

Other (n=6)
